# 645. " Risk of Disease Severity and Disease Outcomes with Serotype-Specific Dengue Virus among Hospitalized Dengue Patients in a Tertiary Infectious Diseases Hospital: A Five-Year Retrospective Study"

**DOI:** 10.1093/ofid/ofad500.709

**Published:** 2023-11-27

**Authors:** Micaela J San Diego, A N A R I A A SAYO, E D N A M EDRADA

**Affiliations:** SAN LAZARO HOSPITAL, PHILIPPINES, PLARIDEL, Bulacan, Philippines; SAN LAZARO HOSPITAL, MANILA, National Capital Region, Philippines; SAN LAZARO HOSPITAL, MANILA, National Capital Region, Philippines

## Abstract

**Background:**

Dengue fever is a significant cause of morbidity throughout the world caused by the dengue virus (DENV). Four antigenically different dengue virus serotypes (DENV-1,2,3, and 4) are known to cause infections in humans (Halsey,2012). Chances for developing dengue hemorrhagic fever-dengue shock syndrome increases significantly with history of previous infection with one of the four serotypes.

**Methods:**

A retrospective study of hospitalized dengue patients in span of five years was done. One-hundred forty three (143) patients aged

>19 years admitted due to fever, positive for NS1 antigen and with DENV serotype results were included. Clinical data were extracted from medical records including demographics, co-morbidities, clinical presentation, laboratory investigations, length of hospital stay and outcome.

**Results:**

Out of the 143 adult dengue patients, the most prevalent serotype was DENV 3 at 64.34% followed by DENV 2 (18.18%), DENV 1 (11.19%), with DENV 4 as the least prevalent serotype (6.29%). In terms of clinical manifestations, the most prevalent symptoms were fever (98.60%), myalgia (78.32%), body malaise (69.23%), headache (59.44%), nausea and vomiting in 42.66%. Majority tested positive for Dengue NS1 (87.94%), with more than half testing positive for Dengue IgG at 54.24%. The adjusted risk for dengue with warning signs for DENV-2 was 5.94, indicating that the risk of dengue with warning signs was 5.94 times higher among those with DENV 2 than those with DENV 1; however, the association was not statistically significant. Results showed that the ARR for serotypes 3 and 4 for all dengue severity categories were not statistically significant (*p*>0.05). The clinical outcome of patients was recovery at 98.60%, with only 1.40% mortality. Comparative analyses indicated that the clinical management and outcomes of the patients did not significantly differ according to dengue virus serotype (*p*>0.05).

**Conclusion:**

This study provided more information on the dengue serotypes, its effect on severity and outcome of the disease. Infecting dengue serotype play an important role in disease severity among adult dengue patients in the Philippines.

**Disclosures:**

**All Authors**: No reported disclosures

